# Immune Response after the Fourth Dose of SARS-CoV-2 mRNA Vaccine Compared to Natural Infection in Three Doses’ Vaccinated Solid Organ Transplant Recipients

**DOI:** 10.3390/v14102299

**Published:** 2022-10-19

**Authors:** Rosalia Busà, Giovanna Russelli, Monica Miele, Maria Concetta Sorrentino, Mariangela Di Bella, Francesca Timoneri, Giuseppina Di Mento, Alessandra Mularoni, Patrizio Vitulo, Pier Giulio Conaldi, Matteo Bulati

**Affiliations:** 1 Research Department, Mediterranean Institute for Transplantation and Advanced Specialized Therapies (IRCCS ISMETT), 90127 Palermo, Italy; 2 Ri.MED Foundation, 90133 Palermo, Italy; 3 Department of Laboratory Medicine and Advanced Biotechnologies, Mediterranean Institute for Transplantation and Advanced Specialized Therapies (IRCCS ISMETT), 90127 Palermo, Italy; 4 Department of Infectious Diseases, Mediterranean Institute for Transplantation and Advanced Specialized Therapies (IRCCS ISMETT), 90127 Palermo, Italy; 5 Department for the Treatment and Study of Cardiothoracic Diseases and Cardiothoracic Transplantation, Mediterranean Institute for Transplantation and Advanced Specialized Therapies (IRCCS ISMETT), 90127 Palermo, Italy

**Keywords:** mRNA vaccine, solid organ transplant recipients, immune response, IgG, IgA, T-cell response, SARS-CoV-2, COVID-19, booster, fourth dose

## Abstract

Solid organ transplant recipients (SOTRs) show higher rates of COVID-19 breakthrough infection than the general population, and nowadays, vaccination is the key preventative strategy. Nonetheless, SOTRs show lower vaccine efficacy for the prevention of severe COVID-19. Moreover, the emergence of new SARS-CoV-2 variants of concern has highlighted the need to improve vaccine-induced immune responses by the administration of repeated booster doses. In this study, we analyzed the humoral and cellular responses in a cohort of 25 SOTRs, including 15 never-infected SOTRs who received the fourth dose of the mRNA vaccine and 10 SOTRs who contracted SARS-CoV-2 infection after the third dose. We analyzed the serum IgG and IgA levels through CLIA or ELISA, respectively, and the Spike-specific T cells by ELISpot assay. We report a significant increase in anti-Spike IgG and no differences in IgA secretion in both groups of patients before and after the booster dose or the natural infection. Still, we show higher IgA levels in recovered SOTRs compared to the fourth dose recipients. Conversely, we show the maintenance of a positive Spike-specific T-cell response in SOTRs who received the fourth dose, which, instead, was significantly increased in SOTRs who contracted the infection. Our results suggest that the booster, either through the fourth dose or natural infection, in vulnerable poor responder SOTRs, improves both humoral and cellular-specific immune responses against SARS-CoV-2.

## 1. Introduction

Solid organ transplant recipients (SOTRs) show higher rates of coronavirus disease 2019 (COVID-19) breakthrough infection than the general vaccinated population [1]. Nowadays, vaccination is a key preventative strategy but is associated with a suboptimal immune response among SOTRs, and accumulating evidence suggests that SOTRs, as opposed to the general population, show lower vaccine efficacy for the prevention of severe COVID-19 [2,3,4,5]. The emergence of new SARS-CoV-2 variants of concern (VOCs), mainly the highly transmissible omicron variants, has highlighted the need to improve vaccine-induced immune responses by the administration of repeated booster doses [6]. Currently, this strategy is debated, and data on the efficacy of repeated boosters is limited. This issue is of particular importance for SOTRs, who are susceptible to the worst effects of SARS-CoV-2 infection [7], and for whom current COVID-19 excess deaths have been described [8]. Despite that, we have previously demonstrated as in SOTRs the third dose of mRNA vaccine induced an improvement in the immune response against SARS-CoV-2 infection [9]. Accordingly, different studies on the efficacy of third vaccination have shown promising results in SOTRs [10,11,12,13]. In SOTRs, insufficient humoral protection against SARS-CoV-2 infection, in combination with a weaker spike-specific T-cell response, is still a relevant concern, and it is supposed that evolving VOCs, such as the Omicron variant, require higher antibody levels compared to the wild-type variant [14,15]. In this context, a fourth vaccine dose was recommended in several countries. Recently, different papers on the efficacy of the fourth booster dose in SOTRs have reported conflicting results. Indeed, Kamar et al. [16] and Alejo et al. [17] reported a slight or no improvement of humoral response after the fourth dose among SOTRs with a weak response after 3 doses of the vaccine. Other authors, instead, indicated an adequate antibodies upsurge in seronegative or low responder patients [18,19]. As an example, Mitchell et al. reported that in 11% of their SOTRs, seronegativity persists, while 61% of patients, who were seronegative after the third dose, seroconverted, and 84% of low responders were boosted to high antibody levels after the fourth dose of the vaccine [19]. In our study, with the aim of assessing the effectiveness of the mRNA vaccine, we followed up on 25 SOTRs, analyzing both their humoral and cellular responses and dividing them into two groups. The first group consisted of 15 never-infected patients who received the fourth dose of the mRNA vaccine at 168 days (range 116–246 days) after the third dose; the second group included 10 SOTRs who, instead, contracted SARS-CoV-2 infection 134 days (range 64–221 days) after the third dose. We report a significant increase in anti-Spike IgG and no differences in anti-Spike IgA secretion in both groups of patients. By contrast, we observed the maintenance of a positive Spike-specific T-cell response in SOTRs who received the fourth dose, while in SOTRs who contracted the infection, we report a significant increase in Spike-specific T-cell response. These results suggest the existence of an immunogenic potential for these poor responder groups. Indeed, the booster, either through the fourth dose or natural infection, in vulnerable patients such as SOTRs, improves both humoral and cellular-specific immune responses against the SARS-CoV-2 virus.

## 2. Materials and Methods

### 2.1. Recruitment and Clinical Sample Collection

We enrolled in ISMETT hospital 25 SOTRs who had received the third dose of the mRNA (Pfizer-BioNTech or Moderna) vaccine between September and November 2021, from whom peripheral blood mononuclear cells (PBMCs) and serum samples were serially collected. All patients were vaccinated with the Pfizer-BioNtech vaccine, except one subject who was vaccinated with the Moderna vaccine. Among these 25 patients, 15 received the fourth dose (TxVac) between March and May 2022, while 10 SOTRs contracted the virus after the third dose of the mRNA vaccine (TxRec) between December 2021 and May 2022. Characteristics of the population are summarized in Table 1. None of the 25 patients included in the study had a history of PCR-confirmed SARS-CoV-2 infection before the third dose of the vaccine. To monitor infection during the overall period of follow-up, apart from a positive nasopharyngeal swab (NPS), we also determined the presence of anti-nucleocapsid (N) antibodies by using both the chemiluminescent assay anti-SARS-CoV-2-N-domain CMIA (IgG and IgM) on the ARCHITECT Quant test (Abbott), and SARS-CoV-2 ELISpot against N-peptides mix (see ELISpot paragraph). We collected blood, PBMCs, and serum samples for the analysis of humoral and cellular responses three weeks after the third dose (T0) for all SOTRs included in the study, and at a median time of two months after the fourth dose or after negativization, for TxVac or TxRec, respectively. The study was approved by the IRCCS ISMETT Institutional Research Review Board (IRRB/00/21) and by the Ethics Committee of ISMETT, and all enrolled patients signed the written informed consent form.

### 2.2. SARS-CoV-2 Anti-Spike IgG and IgA Detection

To detect serum IgG antibodies against S1 and S2 fragments of the Spike protein, we used the chemiluminescent immunoassay (CLIA) LIAISON^®^ Trimeric SARS-CoV-2 S1/S2 IgG (DiaSorin S.p.A., Saluggia, VC, Italy) on the fully automated LIAISON^®^ XL Analyzer (DiaSorin S.p.A., Saluggia, VC, Italy). The concentration of anti-SARS-CoV-2 S1/S2 IgG antibody was expressed as binding antibody unit (BAU) per mL (BAU/mL), and values > 33.8 BAU/mL were considered positive. An enzyme-linked immunoassay (ELISA) was used for the semi-quantitative detection of serum IgA against the S1 fragments of the Spike protein on the fully automated EUROIMMUN Analyzer I (EUROIMMUN, PerkinElmer Company, Hong Kong, China). The anti-SARS-CoV-2 IgA concentrations were expressed as the ratio of the extinction of the sample to that of the calibrator, and the ratio >1.1 were considered positive.

### 2.3. SARS-CoV-2-Specific T Cell ELISpot Assay

To detect IFN-γ-secreting T cells, we used the human IFN-γ ELISpot plus kit (Mabtech AB, Stockholm, Sweden). Briefly, PBMCs of the studied subjects were isolated from whole blood by density gradient centrifugation using a cell preparation tube with sodium citrate (BD Vacutainer^®^ CPT™), according to the manufacturer’s protocol. Then, 2.5 × 10^5^ ± 0.5 × 10^5^ fresh PBMCs/mL (in duplicate) were stimulated for 20–22 h, at 37 °C in a 5% CO_2_ humidified atmosphere. For stimulation we used 1 µg/mL of overlapping peptides spanning the SARS-CoV-2 Spike (Mix I and II, respectively, of 158 and 157 peptides, purity > 90% derived from a peptide scan, 15 mers with 11 aa overlap; PepMix^TM^ SARS-CoV-2 (Spike Glycoprotein), Product Code: PM-WCPV-S-3, Protein ID: P0DTC2, JPT Peptide Technologies, Berlin, Germany) or an N protein peptide pool (purity > 90%, JPT Peptide Technologies, Berlin, Germany). For PBMC culture, we used RPMI 1640 medium (BIOWEST, Nuaillé, France), supplemented with 5% GemCell™ U.S. Origin Human Serum AB (BIOIVT, Westbury, NY, USA) and 1% L-glutamine (Euroclone, Pero, Italy). Unstimulated cells were used as a negative control, while PBMCs were activated through anti-CD3 and CEFX PepMix (a pool of 176 known peptides from various infectious agents, JPT Peptide Technologies, Germany) and were considered as a positive control. The number of SARS-CoV-2-specific IFN-γ-secreting T cells were detected, according to ELISpot guidelines [20], by using an ELISpot Reader (Autoimmun Diagnostika (AID) GmbH, Straßberg, Germany) and determined through the ELISpot Software (AID). To generate normalized reading, we subtracted from the mean of test wells the mean spot counts of negative control wells. Results were presented as spot forming unit (SFUs) per million (SFC/10^6^). The mean value of responses of unstimulated wells plus two standard deviations (SDs) were used to determine the lower limit for indicating a positive response (cutoff = 112 SFC/10^6^ PBMC).

### 2.4. Statistical Analysis

We performed statistical analysis by using GraphPad Prism 9.0 (GraphPad Software, San Diego, CA, USA). Wilcoxon’s matched-pairs nonparametric test, the Mann–Whitney test, Welch’s *t*-test, and one-way ANOVA tests with multiple comparisons were used, according to the type of samples to compare. Correlations were performed using Spearman’s rank correlation coefficient. Statistical significance was determined as * *p* < 0.0332, ** *p* < 0.0021, *** *p* < 0.0002, and **** *p* < 0.0001.

## 3. Results

### 3.1. Characteristics of the Study Population

In this study, 25 SOTRs (16 men, 64%) with a median age of 55 years (IQR, 49–66.5 years), with no history of COVID-19 and a negative SARS-CoV-2 anti-N serology at the time of inclusion, were enrolled. Patients’ baseline characteristics are shown in Table 1. Among 10 SOTRs who contracted the virus after the third dose, only three subjects had severe symptoms and required hospitalization, while the remaining seven subjects had moderate symptoms. These patients are the same as those who were included in our previous study [9], except for two patients who died and two who recovered but refused to continue the study. The immunosuppressive (IS) therapy included calcineurin inhibitors (CNI, tacrolimus) (92%, 23/25 patients), mTOR inhibitors (everolimus) (8%, 2/25 patients), mycophenolate mofetil (MMF) (68%, 17/25 patients), and steroids (56%, 14/25 patients).

### 3.2. Humoral Response Elicited by Fourth Booster Dose or Natural Infection after the Third Dose of the mRNA Vaccine

**Figure 1:**
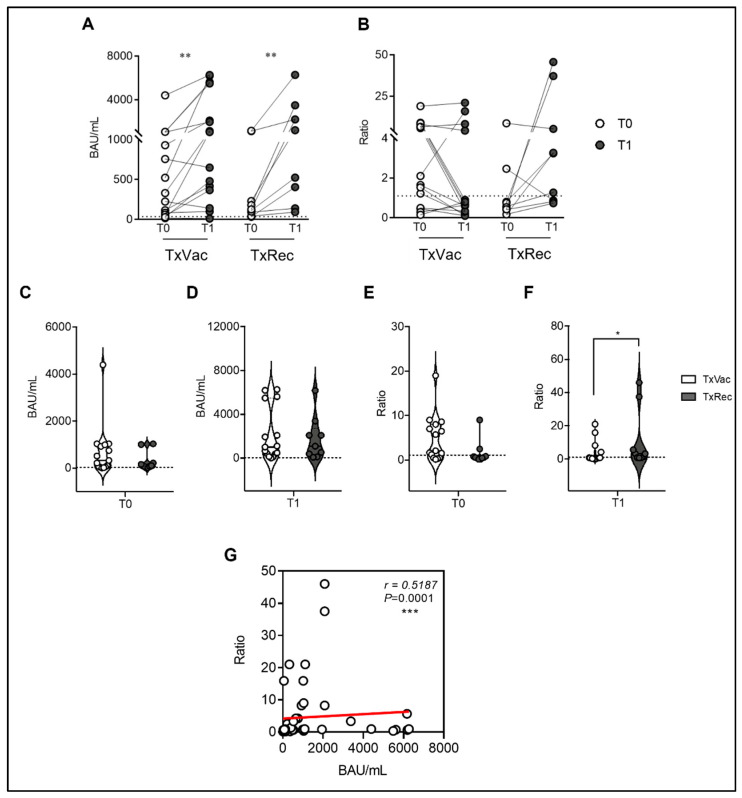
Humoral immune response to SARS-CoV-2 vaccination in SOTRs (*n* = 25) after the third dose of the mRNA vaccine (T0), and alternately, after the administration of the fourth booster dose (*n* = 15), or after the onset of the natural infection (*n* = 10). (**A**) Comparison of anti-SARS-CoV-2 S1/S2 IgG concentration between T0 (white dots) and T1 (grey dots), respectively, after the fourth dose (TxVac) or after natural infection (TxRec). Samples with anti-SARS-CoV-2 S1/S2 IgG concentration >33.8 BAU/mL were considered positive. (**B**) Comparison of anti-SARS-CoV-2 S1 IgA ratio between T0 (white dots) and T1 (grey dots), respectively, after the fourth dose (TxVac) or after the natural infection (TxRec). Ratios ≥ 1.1 were considered positive. Comparison of anti-SARS-CoV-2 S1/S2 IgG at T0 (**C**) and T1 (**D**) between TxVac and TxRec. Comparison of anti-SARS-CoV-2 S1 IgA at T0 (**E**) and T1 (**F**) between TxVac and TxRec. (**G**) Correlation between the total anti-SARS-CoV-2 S1/S2 IgG (BAU/mL) levels against anti-SARS-CoV-2 S1 IgA (Ratio) levels of both groups. The connection lines represent the antibody value of each subject at T0 and T1, while the dotted line corresponds to the threshold. The significances were determined using the Wilcoxon matched-pairs signed-rank test (two-tailed), the Mann–Whitney test, and Spearman’s rank correlation (two-sided); * *p* < 0.0332, ** *p* < 0.0021, *** *p* < 0.0002.

To expand our knowledge of the vaccine immunogenicity in SOTRs, we followed up the anti-SARS-CoV-2 antibody response in a cohort of 25 SOTRs at two different time points. Namely, at T0 after the third dose of mRNA vaccine, and alternately, after the administration of the fourth booster dose (TxVac, *n* = 15), or after the onset of the natural infection (TxRec, *n* = 10). Comparing serum Spike-specific IgG antibody levels before and after the fourth dose (TxVac) of the mRNA vaccine, we found a significant increase (*p* = 0.0015) in the median value of IgG at T1 (threefold change T0/T1), suggesting the efficacy of the fourth booster dose in increasing the IgG humoral response (Figure 1A). Indeed, at T0, the median value of specific IgG was 330.2 BAU/mL (IQR, 59.02–1001; SEM = 283.7), which increased to a median value of 1020 BAU/mL at T1 (IQR, 366.6–5486; SEM = 628.1). Importantly, two patients in this cohort were seronegative for anti-Spike IgG after the third dose and did not seroconvert after the fourth dose, as reported elsewhere [19]. Conversely, we did not observe any significant change in Spike-specific IgA production after the fourth dose (*p* = 0.2293). In fact, as depicted in Figure 1B, the median ratios of serum-specific IgA were 2.1 (IQR, 0.49–8.00; SEM = 1.3) at T0 and 0.64 (IQR, 0.33–4.22; SEM = 1.6) at T1. However, in recovered SOTRs, we report a 10.5-fold significant increase in Spike-specific serum IgG (*p* = 0.0039) at T1 (median = 1089 BAU/mL; IQR, 263.5–2730; SEM = 662.7) compared to T0 (median = 103.4 BAU/mL; IQR, 60.39–417.9; SEM = 125.1) (Figure 1A). In this group, we also observed that two SOTRs, which did not present Spike-specific IgG after the third dose, showed seroconversion after the recovery from natural infection. Furthermore, we show a slight increase in Spike-specific IgA (Figure 1B), although not statistically significant (*p* = 0.2031), at T1 (median ratio = 3.27; IQR, 0.87–21.57; SEM = 5.86) compared to T0 (median ratio = 0.58; IQR, 0.38–1.24; SEM = 0.85). Moreover, we also compared IgG (Figure 1C,D) and IgA levels (Figure 1E,F) for each time point (T0 and T1) between the two cohorts studied (TxVac and TxRec), and we did not find any statistical significance, except for IgA levels at T2, in which we observed a fivefold significant increase in Spike-specific IgA (*p* = 0.026) in TxRec (median = 3.27; IQR,0.87–21.57) compared the TxVac (median = 0.64; IQR, 0.33–4.22). Finally, plotting the data of all IgG versus all IgA values (Figure 1G), for both time points, we found a positive correlation between the oscillation of both immunoglobulin classes (*r* = 0.5187, *p* = 0.0001), suggesting a similar trend in both groups.

### 3.3. SARS-CoV-2-Specific T-Cell Responses Elicited by the Fourth Booster Dose or Natural Infection after the Third Dose of the mRNA Vaccine

**Figure 2:**
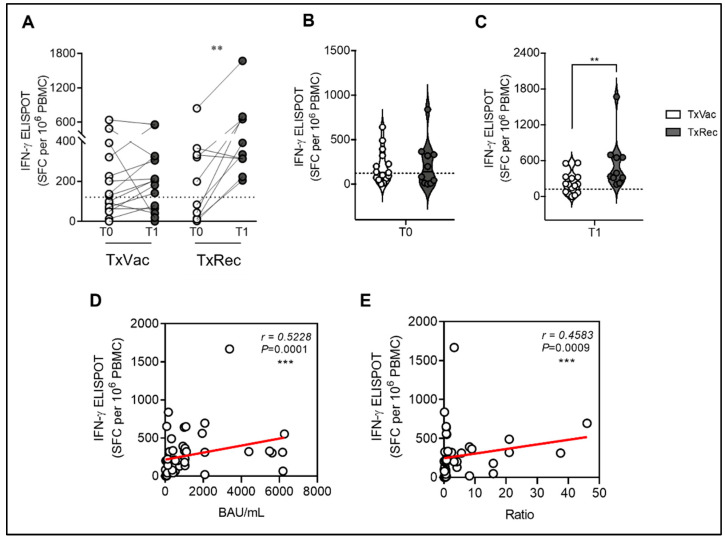
Cellular immune response to SARS-CoV-2 vaccination in SOTRs (*n* = 25) after the third dose of the mRNA vaccine (T0), and alternately, after the administration of the fourth booster dose (*n* = 15), or after the onset of the natural infection (*n* = 10). (**A**) T-cell responses (IFN-γ ELISpot SFC per 10^6^ PBMC) to Spike were compared between T0 (white dots) and T1 (grey dots), respectively after the fourth dose (TxVac) or after natural infection (TxRec). Each dot plot represents the normalized mean spot count from duplicate wells (2.5 ± 0.5 × 10^5^ PBMC/well) for each subject, after subtraction of the spot count of unstimulated cells. IFN-γ ELISpot > 112 SFC/10^6^ PBMC were considered positive. Comparison of T-cell responses (IFN-γ ELISpot SFC per 10^6^ PBMC) to Spike at T0 (**B**) and T1 (**C**) between TxVac and TxRec. (**D**) Correlation of T-cell responses (IFN-γ ELISpot SFC per 10^6^ PBMC) to Spike against anti-SARS-CoV-2 IgG (BAU/mL) levels of both groups. (**E**) Correlation of T-cell responses (IFN-γ ELISpot SFC per 10^6^ PBMC) to Spike against anti-SARS-CoV-2 S1 IgA (ratio) levels of both groups. The connection lines represent the antibody value of each subject at T0 and T1, while the dotted line corresponds to the threshold. The significances were determined using the Wilcoxon matched-pairs signed-rank test (two-tailed), Mann–Whitney test, and Spearman’s rank correlation (two-sided); ** *p* < 0.0021, *** *p* < 0.0002.

To further understand the effect of a “re-stimulation”, either by a fourth booster dose of the mRNA vaccine or through natural infection, on the immune response of three-dose-vaccinated SOTRs, we investigated the T-cell-mediated immunity in both groups of patients. As shown in Figure 2A, in the 15 SOTRs without prior reported SARS-CoV-2 infection (TxVac) who received four doses of the mRNA vaccine, we did not show any significant difference (*p* = 0.3258) between T0 (median = 129 SFC/10^6^ PBMC; IQR, 63.0–129.0; SEM = 48.5) and T1 (median = 203 SFC/10^6^ PBMC; IQR, 65.0–321.0; SEM = 45.2) while maintaining a positive T-cell response. Interestingly, in the cohort of SOTRs who recovered from natural infection (TxRec), we reported a 2.5-fold significant increase (*p* = 0.0098) of Spike-specific T-cell response at T1 (median = 363.0 SFC/10^6^ PBMC; IQR, 289.5–662.5; SEM = 137.6) compared to T0 (median = 140.0 SFC/10^6^ PBMC; IQR, 11.25–339.5; SEM = 82.59). The intriguing result is that the five SOTRs in this cohort, previously non-responders, mounted a positive T-cell response after natural infection. Moreover, we also compared the Spike-specific T-cell response at T0 (Figure 2B) and at T1 (Figure 2C) between the two cohorts studied, finding a twofold significant increase in Spike-specific T-cell response (*p* = 0.0038) in TxRec (median = 363.0; IQR, 289.5–662.5) compared with TxVac (median = 203.0; IQR, 65–321). Moreover, we found a positive correlation between the cellular and the humoral responses for both IgG (*r* = 0.5228 and *p* = 0.0001) (Figure 2D) and IgA (*r* = 0.4583 *p* = 0.0009) (Figure 2E). Moreover, to verify SARS-CoV-2 infection, we stimulated PBMC of both groups of patients with SARS-CoV-2 N-protein. In this case, we obtained a median of 22 SFC/10^6^ PBMC (under cut-off for positive response) in uninfected SOTRs and of 234 SFC/10^6^ PBMC in recovered ones (data not shown).

## 4. Discussion

Despite the prioritization of frail individuals within COVID-19 vaccine protocols, this population, and in particular SOTRs, remains vulnerable to the worst effects of SARS-CoV-2 infection, as high morbidity and mortality have been reported [2,3,4,5]. SOTRs exhibit different responses to SARS-CoV-2 infection or vaccination, and available vaccines elicit lower immune responses compared to the overall population [5]. It has been reported that the third dose of mRNA vaccine induces an improvement of SOTRs’ immune response [9,21,22], but in a subset of patients who had not achieved a response after two doses, it remained at low levels [5], demonstrating that even the third dose of mRNA vaccine results are insufficient in inducing a protective immune response against the virus. For this reason, additional COVID-19 vaccine booster doses could be necessary to improve vaccine efficacy and protect against emerging SARS-CoV-2 variants. For this purpose, it is still not clear as to the real-life effectiveness of a fourth booster dose in SOTRs, as conflicting results have been recently reported [16,17,18,19]. In this paper, we analyzed humoral and cellular immune response in a small cohort of 15 SOTRs, without prior reported SARS-CoV-2 infection, almost two months after the fourth dose of mRNA vaccine. Moreover, we compared this cohort of patients to a second group consisting of 10 SOTR subjects who contracted the infection some months after the third dose. Analyzing the immune response elicited by the third dose, the most relevant data concern the lower levels of serum IgA, which belong to SOTRs who contracted the infection, as previously reported [9]. Indeed, 80% of patients within this group failed to mount an anti-Spike IgA response, a sign of increased susceptibility to infection. Among these patients, only three developed severe symptoms, while the remaining seven developed only mild symptoms and did not need hospitalization, probably due to the positive anti-Spike IgG serology induced by the third dose of the mRNA vaccine. Two months after a negative NPS, the recovered subjects showed a substantial increase in Spike-specific serum IgG and a slight increase in IgA, though not significant, compared to T0. Intriguingly, the two patients who were IgG seronegative after the third dose had seroconversion. Similarly, the counterpart of subjects vaccinated with four doses reported a significant increase in anti-Spike IgG, but the two patients that were seronegative before fourth dose did not seroconvert, as reported by other researchers [19]. Concerning Spike-specific IgA secretion, we did not report any significant differences with respect to T0, but we found that recovered SOTRs showed higher levels compared to the fourth booster dose recipients. Conversely, SOTRs who received the fourth dose, even though remaining positive, did not show any significant difference in Spike-specific T-cell response, while in recovered SOTRs, we report a significant increase in Spike-specific T-cell response. Interestingly, 50% of these subjects who did not show a Spike-specific T-cell response before infection became positive after negativization. These results are in agreement with the current opinion that hybrid immunity (vaccination plus SARS-CoV-2 infection in any order) confers greater protection than immunity elicited by vaccination or COVID-19 unconnectedly [23,24,25,26,27]. Moreover, our results suggest that an immunogenic potential of the mRNA vaccine exists also in fragile subjects. Indeed, the booster, through either the fourth additional dose or natural infection, in vulnerable poor responder patients, such as SOTRs, improves both humoral and cellular-specific immune responses against SARS-CoV-2. The limitations of our study include the small sample size; the heterogeneity in the type of transplanted organs; and, finally, the lack of formal neutralizing antibodies. For this purpose, it has been recently reported that anti-Spike IgG levels over 143 BAU/mL are related to a neutralizing antibody activity against the wild-type virus and the Alpha, Beta, and Gamma variants, while the Delta variant requires higher antibody levels [15]. Concerning the Omicron variant, it is unknown as to the correct protective antibody level, although the degree of protection against the infection and the severe disease progressions increases with rising antibody levels [28]. Nevertheless, in high-risk patients where anti-Spike seronegativity persists, continued preventive measures should remain essential, including passive immune prophylaxis (as the use of monoclonal antibodies) and improved vaccination strategies that elicit a strong and long-lasting immune response.

## Figures and Tables

**Table 1:** Baseline characteristics of SOTRs. ^1^ tacrolimus; ^2^ everolimus; ^3^ *p*-value of [A], [B], and [C] from all patients was not significant (*p*-value between A and B = 0.253, between A and C = 0.303, and between B and C = 0.962). Welch’s *t*-test was used for statistical analyses, and *p* < 0.05 was considered statistically significant. Abbreviations: yr, year; SD, standard deviation; M, male; n, number; MMF, mycophenolate mofetil.

Variable	TxVac (*n* = 15)	TxRec (*n* = 10)	*p*-Value
Age, mean yr (SD)	58 (13)	51 (14)	0.2370
Gender, M (%)	9 (60)	7 (70)	
Type of transplant, *n* (%)			
Kidney	3 (20)	2 (20)	
Lung	6 (40)	4 (40)	
Liver	4 (26.7)	3 (30)	
Heart	2 (13.3)	1 (10)	
Time from transplant, median yr (range)	6 (2–15)	10.5 (2–28)	0.0846
Immunosuppressive treatment, n (%),			
Calcineurin inhibitors ^1^ (3.2 to 14.0 ng/mL) mean ng/mL (SD)	14 (93.3%), 7.06 (2.59)	9 (90%), 7.26 (3.83)	
mTOR inhibitors ^2^ (3.39 to 5.0 ng/mL) mean ng/mL (SD)	2 (13.3%), 4.20 (1.15)	------	
Mycophenolate-mofetil (MMF) (360 to 2000 mg) mean mg (SD)	9 (60%), 826.66 (284.07)	8 (80%), 1125 (353.5)	
Steroids (5 to 14.64 mg) mean mg (SD)	8 (53.3%), 6.49 (3.53)	5 (50%), 6 (2.24)	
Timespan between 3° dose and 4° dose, mean days (range)	168.33 (116–246)	------	
Timespan between 3° dose and COVID-19, mean days (range)	------	134.50 (64–221)	
Timespan between 3° dose/sampling, mean days (range) [A]	52.27 (21–110) ^3^	54.1 (19–98)	0.8655
Timespan between 4° dose/sampling, mean days (range) [B]	65.33 (26–127) ^3^	------	
Timespan between COVID-19/sampling, mean days (range) [C]	------	64.70 (28–141) ^3^	
Comorbidities, *n* (%)			
Diabetes	5 (33.3)	3 (30)	
Obesity	1 (6.67)	2 (20)	
Hypertension	4 (26.7)	5 (50)	
Dyslipidaemia	3 (20)	2 (20)	
Active or previous smoker	4 (26.7)	2 (20)	
Cardiovascular disease	3 (20)	4 (40)	
Kidney disease	1 (6.67)	3 (30)	
Pulmonary disease	2 (13.3)	0	
Gastrointestinal disease	5 (33.3)	2 (20)	
Endocrinal disease	4 (26.7)	0	
History of malignancy	6 (40)	2 (20)	

## Data Availability

The original contributions presented in the study are included in the article. Further inquiries can be directed to the corresponding authors.

## References

[1] Saharia K.K., Anjan S., Streit J., Beekmann S.E., Polgreen P.M., Kuehnert M., Segev D.L., Baddley J.W., Miller R.A., Team E.C.-S. (2022). Clinical characteristics of COVID-19 in solid organ transplant recipients following COVID-19 vaccination: A multicenter case series. Transpl. Infect. Dis. Off. J. Transplant. Soc..

[2] Guarino M., Cossiga V., Esposito I., Furno A., Morisco F. (2022). Effectiveness of SARS-CoV-2 vaccination in liver transplanted patients: The debate is open!. J. Hepatol..

[3] Marinaki S., Adamopoulos S., Degiannis D., Roussos S., Pavlopoulou I.D., Hatzakis A., Boletis I.N. (2021). Immunogenicity of SARS-CoV-2 BNT162b2 vaccine in solid organ transplant recipients. Am. J. Transplant. Off. J. Am. Soc. Transplant. Am. Soc. Transpl. Surg..

[4] Miele M., Busa R., Russelli G., Sorrentino M.C., Di Bella M., Timoneri F., Mularoni A., Panarello G., Vitulo P., Conaldi P.G. (2021). Impaired anti-SARS-CoV-2 humoral and cellular immune response induced by Pfizer-BioNTech BNT162b2 mRNA vaccine in solid organ transplanted patients. Am. J. Transplant. Off. J. Am. Soc. Transplant. Am. Soc. Transpl. Surg..

[5] Sakuraba A., Luna A., Micic D. (2022). A Systematic Review and Meta-Analysis of Serologic Response following Coronavirus Disease 2019 (COVID-19) Vaccination in Solid Organ Transplant Recipients. Viruses.

[6] Kumar D., Hu Q., Samson R., Ferreira V.H., Hall V.G., Ierullo M., Majchrzak-Kita B., Hardy W., Gingras A.C., Humar A. (2022). Neutralization against Omicron variant in transplant recipients after three doses of mRNA vaccine. Am. J. Transplant. Off. J. Am. Soc. Transplant. Am. Soc. Transpl. Surg..

[7] Kittleson M.M., Chambers D.C., Cypel M., Potena L. (2021). COVID-19 in recipients of heart and lung transplantation: Learning from experience. J. Heart Lung Transplant. Off. Publ. Int. Soc. Heart Transplant..

[8] Massie A.B., Werbel W.A., Avery R.K., Po-Yu Chiang T., Snyder J.J., Segev D.L. (2022). Quantifying excess deaths among solid organ transplant recipients in the COVID-19 era. Am. J. Transplant. Off. J. Am. Soc. Transplant. Am. Soc. Transpl. Surg..

[9] Miele M., Busa R., Russelli G., Sorrentino M.C., Di Bella M., Timoneri F., Vitale G., Calzolari E., Vitulo P., Mularoni A. (2022). Analysis of the Specific Immune Response after the Third Dose of mRNA COVID-19 Vaccines in Organ Transplant Recipients: Possible Spike-S1 Reactive IgA Signature in Protection from SARS-CoV-2 Infection. Microorganisms.

[10] Hall V.G., Ferreira V.H., Ku T., Ierullo M., Majchrzak-Kita B., Chaparro C., Selzner N., Schiff J., McDonald M., Tomlinson G. (2021). Randomized Trial of a Third Dose of mRNA-1273 Vaccine in Transplant Recipients. N. Engl. J. Med..

[11] Schrezenmeier E., Rincon-Arevalo H., Stefanski A.L., Potekhin A., Straub-Hohenbleicher H., Choi M., Bachmann F., Pross V., Hammett C., Schrezenmeier H. (2021). B and T Cell Responses after a Third Dose of SARS-CoV-2 Vaccine in Kidney Transplant Recipients. J. Am. Soc. Nephrol..

[12] Stumpf J., Tonnus W., Paliege A., Rettig R., Steglich A., Gembardt F., Kessel F., Kroger H., Arndt P., Sradnick J. (2021). Cellular and Humoral Immune Responses After 3 Doses of BNT162b2 mRNA SARS-CoV-2 Vaccine in Kidney Transplant. Transplantation.

[13] Westhoff T.H., Seibert F.S., Anft M., Blazquez-Navarro A., Skrzypczyk S., Zgoura P., Meister T.L., Pfaender S., Stumpf J., Hugo C. (2021). A third vaccine dose substantially improves humoral and cellular SARS-CoV-2 immunity in renal transplant recipients with primary humoral nonresponse. Kidney Int..

[14] Hoffmann M., Kruger N., Schulz S., Cossmann A., Rocha C., Kempf A., Nehlmeier I., Graichen L., Moldenhauer A.S., Winkler M.S. (2022). The Omicron variant is highly resistant against antibody-mediated neutralization: Implications for control of the COVID-19 pandemic. Cell.

[15] Planas D., Veyer D., Baidaliuk A., Staropoli I., Guivel-Benhassine F., Rajah M.M., Planchais C., Porrot F., Robillard N., Puech J. (2021). Reduced sensitivity of SARS-CoV-2 variant Delta to antibody neutralization. Nature.

[16] Kamar N., Abravanel F., Marion O., Romieu-Mourez R., Couat C., Del Bello A., Izopet J. (2021). Assessment of 4 Doses of SARS-CoV-2 Messenger RNA-Based Vaccine in Recipients of a Solid Organ Transplant. JAMA Netw. Open.

[17] Alejo J.L., Mitchell J., Chiang T.P., Abedon A.T., Boyarsky B.J., Avery R.K., Tobian A.A.R., Levan M.L., Massie A.B., Garonzik-Wang J.M. (2021). Antibody Response to a Fourth Dose of a SARS-CoV-2 Vaccine in Solid Organ Transplant Recipients: A Case Series. Transplantation.

[18] Caillard S., Thaunat O., Benotmane I., Masset C., Blancho G. (2022). Antibody Response to a Fourth Messenger RNA COVID-19 Vaccine Dose in Kidney Transplant Recipients: A Case Series. Ann. Intern. Med..

[19] Mitchell J., Alejo J.L., Chiang T.P.Y., Kim J., Chang A., Abedon A.T., Avery R.K., Tobian A.A.R., Massie A.B., Levan M.L. (2022). Antibody Response to a Fourth Dose of SARS-CoV-2 Vaccine in Solid Organ Transplant Recipients: An Update. Transplantation.

[20] Janetzki S., Price L., Schroeder H., Britten C.M., Welters M.J., Hoos A. (2015). Guidelines for the automated evaluation of Elispot assays. Nature protocols.

[21] Saharia K.K., Husson J.S., Niederhaus S.V., Iraguha T., Avila S.V., Yoo Y.J., Hardy N.M., Fan X., Omili D., Crane A. (2022). Humoral immunity against SARS-CoV-2 variants including omicron in solid organ transplant recipients after three doses of a COVID-19 mRNA vaccine. Clin. Transl. Immunol..

[22] Tauzin A., Beaudoin-Bussieres G., Gong S.Y., Chatterjee D., Gendron-Lepage G., Bourassa C., Goyette G., Racine N., Khrifi Z., Turgeon J. (2022). Humoral immune responses against SARS-CoV-2 Spike variants after mRNA vaccination in solid organ transplant recipients. iScience.

[23] Bowman K.A., Stein D., Shin S., Ferbas K.G., Tobin N.H., Mann C., Fischinger S., Ollmann Saphire E., Lauffenburger D., Rimoin A.W. (2022). Hybrid Immunity Shifts the Fc-Effector Quality of SARS-CoV-2 mRNA Vaccine-Induced Immunity. mBio.

[24] Ferrara P., Ponticelli D., Magliuolo R., Borrelli M., Schiavone B., Mantovani L.G. (2022). Time-Varying Effect of Hybrid Immunity on the Risk of Breakthrough Infection after Booster Dose of mRNA COVID-19 Vaccine: The MOSAICO Study. Vaccines.

[25] Kajanova I., Grossmannova K., Jelenska L., Lukacikova L., Radikova Z., Knutova N., Nahlikova J., Belisova M., Pastorekova S., Kopacek J. (2022). Seroprevalence of SARS-CoV-2 antibodies in the county town of Slovakia-A pilot study from the Trencin city. Acta Virol..

[26] Pusnik J., Konig J., Mai K., Richter E., Zorn J., Proksch H., Schulte B., Alter G., Streeck H. (2022). Persistent Maintenance of Intermediate Memory B Cells Following SARS-CoV-2 Infection and Vaccination Recall Response. J. Virol..

[27] Suryawanshi R., Ott M. (2022). SARS-CoV-2 hybrid immunity: Silver bullet or silver lining?. Nat. Reviews. Immunol..

[28] Khoury D.S., Cromer D., Reynaldi A., Schlub T.E., Wheatley A.K., Juno J.A., Subbarao K., Kent S.J., Triccas J.A., Davenport M.P. (2021). Neutralizing antibody levels are highly predictive of immune protection from symptomatic SARS-CoV-2 infection. Nat. Med..

